# Reminiscence Therapy as a Potential Method to Improve Psychological Health and Quality of Life in Elderly Hepatocellular Carcinoma Patients: A Randomized, Controlled Trial

**DOI:** 10.3389/fsurg.2022.873843

**Published:** 2022-08-02

**Authors:** Teng Li, Bin Li, Lu Tan, Bo Lv

**Affiliations:** Department of Hepatobiliary and Pancreatic Surgery, The Central Hospital of Wuhan, Tongji Medical College, Huazhong University of Science and Technology, Wuhan, China

**Keywords:** reminiscence therapy, hepatocellular carcinoma, elderly, anxiety and depression, quality of life

## Abstract

**Background:**

Reminiscence therapy mitigates psychological issues and improves the quality of life of cancer survivors. However, its role in elderly patients with hepatocellular carcinoma (HCC) is unclear. Thus, we aimed to detect the effect of reminiscence therapy on anxiety, depression, and the quality of life of elderly patients with HCC.

**Methods:**

In total, 106 elderly patients with HCC after resection were randomized in a 1:1 ratio to the reminiscence therapy group (*N* = 54) and control care group (*N* = 52) and then received intervention for 12 months. Anxiety and depression were evaluated by the Hospital Anxiety and Depression Scale (HADS) at baseline [Month (M) 0], 3 months (M3), 6 months (M6), 9 months (M9), and 12 months (M12). Meanwhile, quality of life was assessed using the European Organization for Research and Treatment of Cancer quality of life Questionnaire—Core 30 (QLQ-C30) at M0, M6, and M12.

**Results:**

The HADS for anxiety score at M9 (6.8 ± 2.3 vs. 7.8 ± 2.4, *P* = 0.039) and M12 (6.6 ± 2.4 vs. 7.8 ± 2.6, *P* = 0.013) and the anxiety proportion at M12 (27.8% vs. 46.2%, *P* = 0.050) were reduced in the reminiscence therapy group compared with those in the control care group. Moreover, the HADS for depression score declined at M9 (6.6 ± 2.0 vs. 7.5 ± 2.2, *P* = 0.025) and M12 (6.3 ± 2.3 vs. 7.7 ± 2.6, *P* = 0.005), but the proportion of those with depression was not different at each visit (*P* > 0.05) in the reminiscence therapy group compared with that of the control care group. In addition, the QLQ-C30 global health status score increased at M6 (71.3 ± 12.8 vs. 66.3 ± 12.9, *P* = 0.048) and M12 (74.5 ± 12.9 vs. 68.2 ± 13.3, *P* = 0.014) in the reminiscence therapy group compared to that in the control care group.

**Conclusion:**

Reminiscence therapy effectively mitigates anxiety and depression and improves the quality of life of elderly patients with HCC.

## Introduction

Hepatocellular carcinoma (HCC) is not only one of the most common cancers but also the second most frequent cause of cancer-related deaths worldwide (1–3). Recently, it has been reported that the proportion of elderly patients with HCC is increasing; meanwhile, more patients are diagnosed at an early stage, partly thanks to the advancement in screening programs (4, 5). However, the general prognosis of elderly patients with HCC is hardly satisfactory, even after surgical resection. Furthermore, a certain proportion of elderly patients with HCC suffer from psychological stress, fear of recurrence, and economic burden, which can lead to anxiety, depression, and reduced quality of life (6–8). Hence, establishing effective intervention programs for elderly patients with HCC to manage their mental health and improve their quality of life is crucial to improving their prognosis.

Reminiscence therapy is a common treatment method for dementia patients that involves encouraging them to share their memories and past experiences, which could evoke memories and improve psychological well-being (9–11). Recently, reminiscence therapy has been adopted to improve the psychological health and quality of life of diverse post-operative cancer patients (12–14). For instance, a reminiscence therapy-based care program mitigated anxiety and depression in post-operative non-small-cell lung cancer (NSCLC) patients (13); meanwhile, it also reduced the levels of anxiety and depression and improved satisfaction in glioma patients after tumor resection (15). However, the effect of reminiscence therapy on psychological health and the quality of life in elderly patients with HCC remains obscure.

We conducted a randomized controlled trial to evaluate the effect of reminiscence therapy on anxiety, depression, and the quality of life in elderly HCC patients post-surgical resection.

## Methods

### Patients

Elderly patients with HCC who underwent surgical resection in our hospital between April 2017 and May 2019 were consecutively recruited in this randomized controlled study. The inclusion criteria were the following: the patients (1) had a diagnosis of HCC; (2) were aged between 60 and 80; (3) had China Liver Cancer (CNLC) stage Ia to IIb and suitable for surgical resection; (4) were without vascular invasion; (5) had normal cognitive function and the ability to complete a study assessment form; and (6) were willing to actively cooperate with study evaluation. The exclusion criteria were the following: the patient (1) had a clinical diagnosis of anxiety or depression before enrollment in the study; (2) had a known psychological illness; (3) had difficulty communicating with others; and (4) was accompanied by other malignancies. This study was permitted by the Institutional Review Board of The Central Hospital of Wuhan, Tongji Medical College, Huazhong University of Science and Technology; and registered at the Chinese Clinical Trial Registry with registration number ChiCTR2200058975. All patients provided informed consent.

### Grouping

After their eligibility was confirmed, patients were given an opaque envelope with the patient enrollment number on the cover. The grouping information was sealed in the envelope, then, the patients were randomly divided into a reminiscence therapy group and a control care group depending on the grouping information corresponding to the enrollment number. The random group information was generated using the block randomization method with a 1:1 ratio and a block size of 6.

### Reminiscence Therapy

Patients in the reminiscence therapy group were given reminiscence therapy, starting from the first month after they were discharged from the hospital. Reminiscence therapy was given in the rehabilitation center of our hospital for a total of 12 months, and was conducted in the form of a communication meeting once a month. Each communication meeting (120 min) was organized around one of the following 12 themes: (1) introducing yourself and your family; (2) sharing the memories of childhood; (3) recalling stories from your school days; (4) sharing the memories of marriage; (5) sharing unique customs of your hometown; (6) sharing your work experiences; (7) sharing an interesting trip; (8) sharing an event that affected the course of your life; (9) sharing personal hobbies; (10) sharing your favorite heroes and their legends; (11) individual talent show; and (12) review and farewell. The reminiscence therapy was given in a quiet and comfortable environment with a relaxed atmosphere. Every eight to nine patients formed a subgroup, and the patients sat in a circle within a subgroup. During the communication meeting, the researchers were responsible for leading the communication meeting, controlling the progress of the communication, adjusting the atmosphere of the scene, encouraging the patient to recall the past, and guiding patients to actively participate in the communication and sharing. Patients were advised to bring some materials, such as records, documents, photos, videos, or diaries, to help them better recall their past and share their stories during the communication meeting. In addition, in each communication meeting, the researchers would communicate with patients about HCC treatment, care, and recovery and give them advice.

### Control Care

Patients in the control care group were also invited to return to the hospital monthly for counseling and to communicate with doctors about HCC treatment, care, and recovery after hospital discharge. Each counseling and communication session lasted for 120 min. No other scheduled intervention was performed.

### Outcome Assessment

The Hospital Anxiety and Depression Scale (HADS) was employed to assess the anxiety and depression of patients at baseline (M0), 3 months (M3), 6 months (M6), 9 months (M9), and 12 months (M12) from the start of the intervention. The HADS for anxiety (HADS-A) score and the HADS for depression (HADS-D) score were calculated, and a cutoff value of 8 was used to define the anxiety patients (HADS-A score ≥8) and depression patients (HADS-D score ≥8) (16). The quality of life of patients was evaluated at M0, M6, and M12 using the European Organization for Research and Treatment of Cancer quality of life Questionnaire-Core 30 (QLQ-C30, Version 3). The QLQ-C30 global health status, function, and symptom scores (ranging from 0 to 100) were calculated severally. A higher QLQ-C30 overall health status score or a function score was associated with better overall health status or physical function, while a higher QLQ-C30 symptom score indicated a worse health status (17).

### Sample Size Calculation

In the present study, the HADS-D score at M12 was set as the primary outcome; hence, the sample size was calculated based on the HADS-D score at M12. Referring to a previous study (13), we hypothesized that the mean HADS-D score at M12 in each group was 6.0 ± 2.0 in the reminiscence therapy group and 7.5 ± 2.0 in the control care group. To detect a significant difference, the power (beta) was set as 90% and the type I error (alpha) was set as 0.05. As a consequence, the required sample size was 39 in each group. Furthermore, the dropout rate was set as 25%, which needed 106 patients.

### Statistical Analysis

Data analysis was based on the intention-to-treat (ITT) principle, with all patients included in the analysis. Student’s *t* test, Wilcoxon’s rank-sum test, Chi-square test, and a two-way repeated-measures analysis of variance (ANOVA) were employed to analyse the difference between the two groups. A two-sided *P*-value of less than 0.05 was used to define the statistical significance. SPSS 21.0 software (IBM Corp., Armonk, New York, USA) and GraphPad Prism 6.01 software (GraphPad Software Inc., San Diego, CA, USA) were applied for analysis.

## Results

### Study Flow

**Figure 1:**
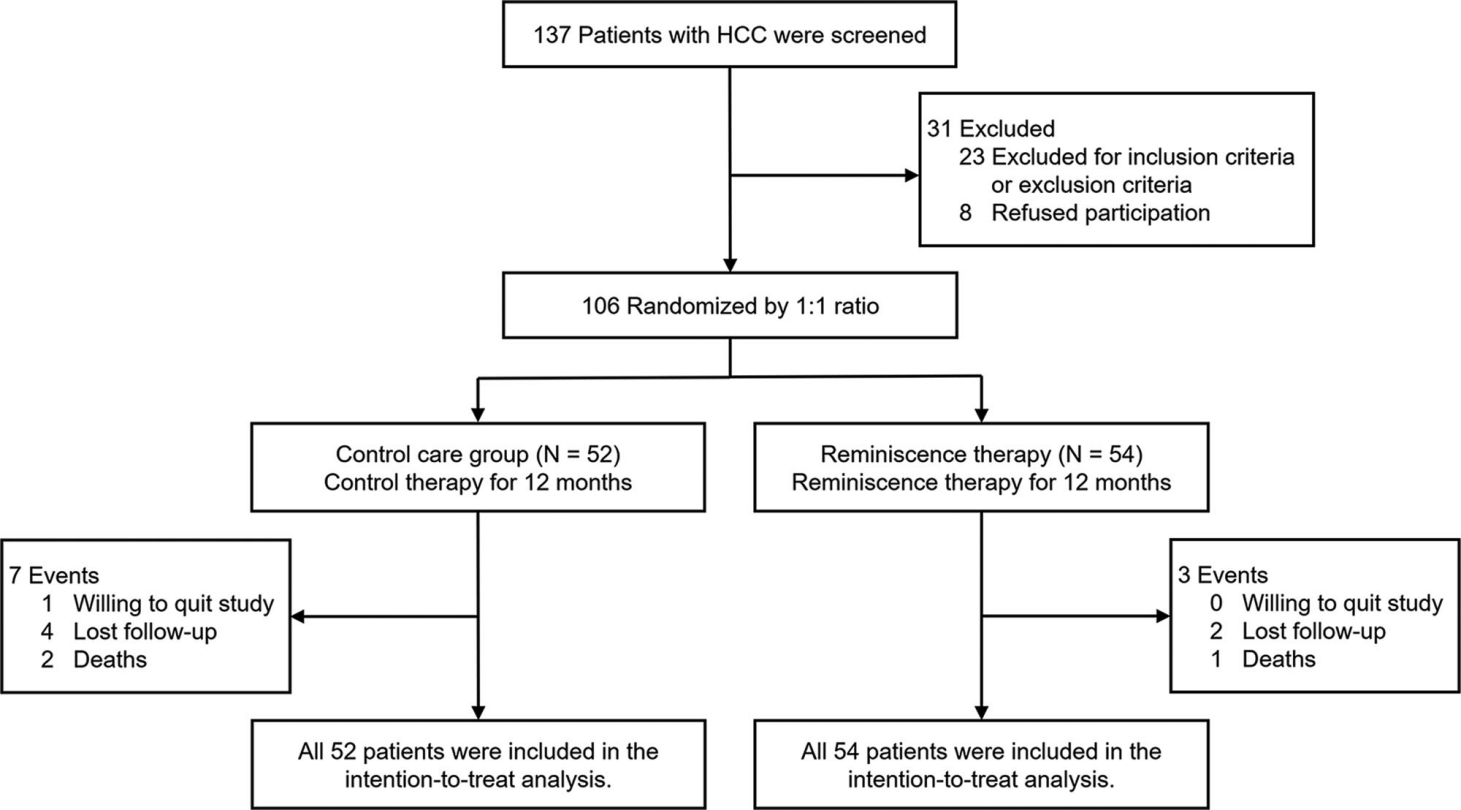
Study flow of the current research.

In total, 137 elderly patients with HCC were screened in the current study; then, 31 of them were excluded, including 23 patients who did not meet the inclusion criteria or who met the exclusion criteria and 8 patients who refused participation. The remaining 106 patients were randomized in a 1:1 ratio into a control care group (*N* = 52), which received monthly usual care including counseling and communicating with a doctor about HCC treatment, care, and recovery, and a reminiscence therapy group (*N* = 54) which received monthly reminiscence therapy and usual care. Subsequently, all patients were monitored for 12 months. In the control care group, one patient quit the study by his/her own will, four patients did not attend follow-up appointments, and two patients died. In the reminiscence therapy group, two patients did not attend follow-up appointments and one patient died. All 106 patients were included in the intention-to-treat analysis (Figure 1).

### Baseline Characteristics

The patients had a mean age of 68.7 ± 5.5 years in the control care group and 68.0 ± 5.4 years in the reminiscence therapy group. There were 6 (11.5%) females and 46 (88.5%) males in the control care group and 8 (14.8%) females and 46 (85.2%) males in the reminiscence therapy group. No difference in the characteristics (including demographics, underlying diseases, disease features, and biochemical indexes) of patients was found between the control care group and the reminiscence therapy group (all *P*’s* *> 0.05). The detailed clinical characteristics of the analyzed patients are presented in Table 1.

**Table 1:** Characteristics of patients.

Items	Control care (*N* = 52)	Reminiscence therapy (*N* = 54)	Statistic (*t*/*χ^2^*/*Z*)	*P* value
Demographics				
Age (years), mean ± SD	68.7 ± 5.5	68.0 ± 5.4	0.672	0.503
Gender, *n* (%)			0.248	0.618
Female	6 (11.5)	8 (14.8)		
Male	46 (88.5)	46 (85.2)		
Marital status, *n* (%)			0.306	0.580
Married	16 (30.8)	14 (25.9)		
Single/divorced/widowed	36 (69.2)	40 (74.1)		
Employment status before surgery, *n* (%)			0.004	0.953
Employed	5 (9.6)	4 (7.4)		
Unemployed	47 (90.4)	50 (92.6)		
Level of education, *n* (%)			0.709	0.871
Primary school or less	6 (11.5)	5 (9.3)		
High school	26 (50.0)	24 (44.4)		
Undergraduate	14 (26.9)	17 (31.5)		
Graduate or above	6 (11.5)	8 (14.8)		
Place of residence, *n* (%)			0.837	0.360
Rural	9 (17.3)	6 (11.1)		
Urban	43 (82.7)	48 (88.9)		
History of smoke, *n* (%)			1.329	0.514
Never	28 (53.8)	24 (44.4)		
Former	14 (26.9)	20 (37.0)		
Current	10 (19.2)	10 (18.5)		
Underlying diseases				
History of HB, *n* (%)	37 (71.2)	39 (72.2)	0.015	0.903
History of liver cirrhosis, *n* (%)	34 (65.4)	34 (63.0)	0.068	0.795
Hypertension, *n* (%)	22 (42.3)	25 (46.3)	0.171	0.679
Hyperlipidemia, *n* (%)	16 (30.8)	19 (35.2)	0.234	0.629
Diabetes, *n* (%)	9 (17.3)	8 (14.8)	0.122	0.727
Disease features				
ECOG PS score, *n* (%)			0.144	0.704
0	42 (80.8)	42 (77.8)		
1	10 (19.2)	12 (22.2)		
Child-Pugh class, *n* (%)			0.012	0.912
A	39 (75.0)	41 (75.9)		
B	13 (25.0)	13 (24.1)		
Tumor nodule number, *n* (%)			1.039	0.308
Unifocal	33 (63.5)	29 (53.7)		
Multifocal	19 (36.5)	25 (46.3)		
Largest tumor size, *n* (%)			<0.001	0.988
<5 cm	24 (46.2)	25 (46.3)		
≥5 cm	28 (53.8)	29 (53.7)		
Vascular invasion, *n* (%)			<0.001	>0.999
No	52 (100.0)	54 (100.0)		
Yes	0 (0.0)	0 (0.0)		
BCLC stage, *n* (%)			−1.584	0.113
0/A	30 (57.7)	21 (38.9)		
B	12 (23.1)	21 (38.9)		
C	10 (19.2)	12 (22.2)		
CNLC stage, *n* (%)			−1.349	0.177
Ia	10 (19.2)	10 (18.5)		
Ib	26 (50.0)	19 (35.2)		
IIa	13 (25.0)	18 (33.3)		
IIb	3 (5.8)	7 (13.0)		
Biochemical indexes				
ALT (U/L), median (IQR)	28.9 (18.4–43.9)	29.3 (23.7–45.2)	−0.531	0.596
AST (U/L), median (IQR)	36.4 (25.4–49.5)	39.1 (27.0–55.1)	−0.638	0.523
CEA (ng/mL), median (IQR)	5.0 (3.6–8.5)	4.1 (3.0–6.9)	−1.282	0.200
CA199 (u/mL), median (IQR)	28.9 (17.2–53.7)	30.0 (14.3–74.7)	−0.219	0.826
AFP (ng/mL), median (IQR)	66.9 (14.0–757.9)	27.6 (7.5–996.4)	−1.180	0.238

*SD, standard deviation; HB, hepatitis B; ECOG PS, Eastern Cooperative Oncology Group Performance Status; BCLC, Barcelona Clinic Liver Cancer; CNLC, China liver cancer; ALT, alanine aminotransferase; IQR, interquartile range; AST, aspartate aminotransferase; CEA, carcinoembryonic Antigen; CA199, cancer antigen 199; AFP, alpha fetoprotein.*

### Anxiety Evaluation

**Figure 2:**
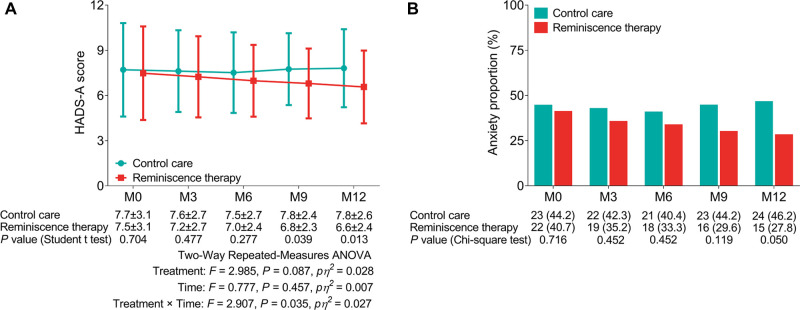
Comparison of anxiety between the reminiscence therapy group and the control care group. Comparison of the HADS-A score (**A**) and anxiety proportion (**B**) between groups at each visit point.

The HADS-A score was lower in the reminiscence therapy group than in the control care group at M9 (6.8 ± 2.3 vs. 7.8 ± 2.4, *P* = 0.039) and M12 (6.6 ± 2.4 vs. 7.8 ± 2.6, *P* = 0.013), while no difference was found in the HADS-A score between the two groups at M0, M3, or M6 (all *P*’s * *> 0.05). Further two-way repeated-measures ANOVA also revealed that reminiscence therapy reduced the HADS-A score (time×treatment: *F* = 2.907, *P* = 0.035, *pη^2^* = 0.027) (Figure 2A). Meanwhile, the proportion of those with anxiety in the reminiscence therapy group was lower than in the control care group at M12 (27.8% vs. 46.2%, *P* = 0.050); however, there was no difference between the two groups at other visit points (all *P*’s * *> 0.05) (Figure 2B).

### Depression Evaluation

**Figure 3:**
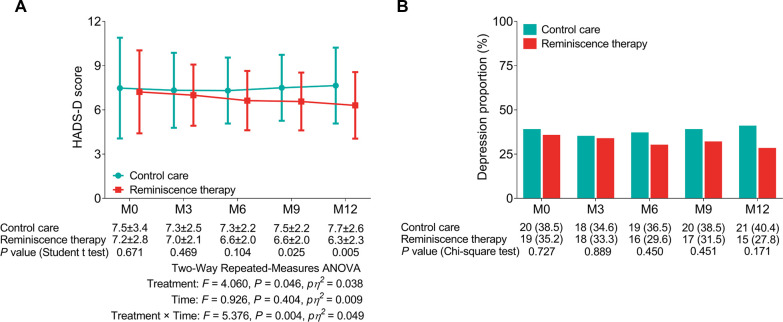
Comparison of depression between the reminiscence therapy group and the control care group. Comparison of the HADS-D score (**A**) and depression proportion (**B**) between groups at each visit point.

The HADS-D score declined in the reminiscence therapy group compared with that in the control care group at M9 (6.6 ± 2.0 vs. 7.5 ± 2.2, *P* = 0.025) and M12 (6.3 ± 2.3 vs. 7.7 ± 2.6, *P* = 0.005), while the HADS-D score was not different between the two groups at M0, M3, M6, or M9 (all *P*’s * *> 0.05). After being adjusted by two-way repeated-measures ANOVA, reminiscence therapy decreased the HADS-D score (time×treatment: *F* = 5.376, *P* = 0.004, *pη^2^* = 0.049) (Figure 3A). In addition, no difference in the proportion of patients with depression was observed between the control care group and the reminiscence therapy group at each visit point (all *P*’s * *> 0.05) (Figure 3B).

### Quality of Life Evaluation

**Figure 4:**
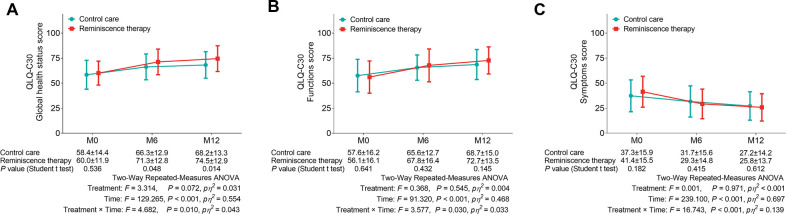
Comparison of the quality of life between the reminiscence therapy group and the control care group. Comparison of the QLQ-C30 global health status score (**A**), QLQ-C30 function score (**B**), and QLQ-C30 sympto score (**C**) between groups at each visit point.

As to the QLQ-C30 global health status score, an increase was recorded in the reminiscence therapy group compared to the control care group at M6 (71.3 ± 12.8 vs. 66.3 ± 12.9, *P* = 0.048) and M12 (74.5 ± 12.9 vs. 68.2 ± 13.3, *P* = 0.014). However, no difference was observed between the two groups at M0 (60.0 ± 11.9 vs. 58.4 ± 14.4, *P* = 0.536) (Figure 4A). Additionally, there were no differences in the QLQ-C30 function score or the QLQ-C30 symptom score between the control care group and the reminiscence therapy group at all visit points (all *P*’s * *> 0.05) (Figures 4B, C). However, two-way repeated-measures ANOVA revealed that reminiscence therapy increased the QLQ-C30 global health status score and function score but decreased the symptom score.

### Subgroup Analysis of Outcomes at M12 of the BCLC Stage and CNLC Stage

In patients with BCLC stage 0/A, the HADS-A score (*P* = 0.033) and HADS-D score (*P* = 0.003) were reduced, while the QLQ-C30 global health status score (*P* = 0.028) was enhanced in the reminiscence therapy group compared with that in the control care group. In patients with BCLC stage B/C, a decrease in HADS-A score (*P* = 0.042) and HADS-D score (*P* = 0.035), as well as an increase in QLQ-C30 functions score (*P* = 0.044), was observed in the reminiscence therapy group compared with that in the control care group (Table 2).

**Table 2:** Subgroup analyses by BCLC stage.

Characteristics	BCLC stage 0/A (*n* = 51)				BCLC stage B/C (*n* = 55)			
	Control care (*n* = 30)	Reminiscence therapy (*n* = 21)	Statistic (*t*/*χ^2^*)	*P* value	Control care (*n* = 22)	Reminiscence therapy (*n* = 33)	Statistic (*t*/*χ^2^*)	*P* value
Outcomes at M12								
HADS-A score, mean ± SD	7.3 ± 1.9	6.0 ± 2.2	−2.193	0.033	8.5 ± 3.2	6.9 ± 2.5	−2.079	0.042
Anxiety proportion, *n* (%)	11 (36.7)	3 (14.3)	3.107	0.078	13 (59.1)	12 (36.4)	2.750	0.097
HADS-D score, mean ± SD	7.0 ± 2.1	5.3 ± 1.8	−3.077	0.003	8.5 ± 3.0	7.0 ± 2.3	−2.168	0.035
Depression proportion, *n* (%)	10 (33.3)	3 (14.3)	2.360	0.125	11 (50.0)	12 (36.4)	1.009	0.315
QLQ-C30, mean ± SD								
Global health status score	70.5 ± 12.5	77.4 ± 9.2	2.270	0.028	65.1 ± 13.9	72.7 ± 14.6	1.927	0.059
Functions score	72.1 ± 14.8	74.6 ± 14.7	0.593	0.556	63.9 ± 14.3	71.5 ± 12.8	2.063	0.044
Symptoms score	22.9 ± 12.7	24.7 ± 14.0	0.482	0.632	33.0 ± 14.4	26.5 ± 13.6	−1.701	0.095

*BCLC, Barcelona Clinic Liver Cancer; M12, month 12; HADS-A, Hospital Anxiety and Depression Scale for anxiety; SD, standard deviation; HADS-D, Hospital Anxiety and Depression Scale for depression; QLQ-C30, Quality of Life Questionnaire—Core 30.*

In patients with CNLC stage I, the HADS-D score (*P* = 0.012) was lower and the QLQ-C30 global health status score (*P* = 0.019) washigher in the reminiscence therapy group compared with that of the control care group. In patients with CNLC stage II, the HADS-A score (*P* = 0.012) was lower, but the QLQ-C30 function score (*P* = 0.032) was higher in the reminiscence therapy group compared with that of the control care group (Table 3).

**Table 3:** Subgroup analyses by CNLC stage.

Characteristics	CNLC stage I (*n* = 65)				CNLC stage II (*n* = 41)			
	Control care (*n* = 36)	Reminiscence therapy (*n* = 29)	Statistic (*t*/*χ^2^*)	*P* value	Control care (*n* = 16)	Reminiscence therapy (*n* = 25)	Statistic (*t*/*χ^2^*)	*P* value
Outcomes at M12								
HADS-A score, mean ± SD	7.3 ± 2.0	6.3 ± 2.4	−1.793	0.078	9.1 ± 3.4	6.9 ± 2.4	−2.371	0.023
Anxiety proportion, *n* (%)	14 (38.9)	6 (20.7)	2.497	0.114	10 (62.5)	9 (36.0)	2.755	0.097
HADS-D score, mean ± SD	7.3 ± 2.5	5.8 ± 2.0	−2.592	0.012	8.5 ± 2.7	6.9 ± 2.4	−1.982	0.055
Depression proportion, *n* (%)	13 (36.1)	7 (24.1)	1.081	0.298	8 (50.0)	8 (32.0)	1.328	0.249
QLQ-C30, mean ± SD								
Global health status score	70.7 ± 12.2	77.2 ± 9.7	2.402	0.019	62.5 ± 14.1	71.3 ± 15.4	1.848	0.072
Functions score	72.1 ± 14.4	74.9 ± 14.2	0.791	0.432	60.9 ± 13.7	70.2 ± 12.5	2.230	0.032
Symptoms score	23.4 ± 12.1	24.0 ± 13.4	0.184	0.855	35.6 ± 15.2	27.9 ± 13.9	−1.677	0.101

*CNLC, China liver cancer; M12, month 12; HADS-A, Hospital Anxiety and Depression Scale for anxiety; SD, standard deviation; HADS-D, Hospital Anxiety and Depression Scale for depression; QLQ-C30, Quality of Life Questionnaire—Core 30.*

## Discussion

Most cancer survivors suffer from many types of stress, such as cancer-related fears, pain, and high economic burden during treatment (18, 19), which then cause anxiety and depression and may lead to low compliance and even suicide (20–23). Thus, establishing an effective care program to alleviate anxiety and depression is crucial. Reminiscence therapy has been shown to relieve anxiety and depression in cancer survivors, especially in elderly patients. For instance, reminiscence therapy care is more effective in improving anxiety and depression in colorectal patients aged ≥65 years (24), and reminiscence therapy reduces anxiety and depression in post-operative cervical cancer patients, especially in elderly patients (25), while the role of reminiscence therapy in elderly patients with HCC is unclear. Our results showed that reminiscence therapy reduced anxiety and depression to a certain extent in elderly patients with HCC, which is consistent with previous studies (24, 25). The explanation might be that (1) listening and sharing personal experiences might help patients to better understand their current situation, which encourages them to have a positive attitude to face life, and thereby alleviates anxiety and depression; and (2) talking with others improves their communication skills and helps them to create new relationships, which could relieve anxiety and depression in elderly patients with HCC.

The overwhelming majority of cancer patients are facing the quality-of-life reduction due to long-term chemotherapy, losing physical function, and many comorbidities (26, 27). Reminiscence therapy is recently applied to improve the quality of life in cancer survivors such as postoperative gastric cancer, NSCLC, and cervical cancer patients (13, 14, 25). However, the effect of reminiscence therapy on elderly patients with HCC remains obscure. Therefore, we conducted this study, and our findings showed that reminiscence therapy improved their quality of life. Possible reasons for our results might be that (1) reminiscence therapy alleviated anxiety and depression in elderly patients with HCC (mentioned above), which might directly relieve psychological pressure and enhance motivation to improve their quality of life; and (2) reminiscence therapy, including regular communication with other patients, might reduce their loneliness and increase their quality of life.

In addition, we detected the effect of reminiscence therapy on mental health and the quality of life in HCC patients with different BCLC stages and CNLC stages, which showed that reminiscence therapy could mitigate anxiety and depression to some extent in every subgroup, while improving patients’ quality of life. The reasons might be that (1) reminiscence therapy showed an outstanding effect in increasing patients’ self-confidence and social ability, as well as decreasing negative emotion, and mitigating anxiety and depression up to a point for elderly patients with HCC at all BCLC and CNLC stages; and (2) reminiscence therapy mainly affected anxiety, depression, and quality of life by encouraging patients to recall their experiences and positive memory to regulate their mood, which might not be affected by the disease-stage classification; thus, reminiscence therapy could mediate anxiety, depression, and the quality of life in elderly patients with HCC regardless of disease stages.

In our study, we designed reminiscence therapy and control care for elderly patients with HCC; among these, the control care group also received routine communication and counseling from a doctor at the same time and frequency as the reminiscence therapy group. Hence, our study eliminated the effects of counseling and communication, which differentiated it from other research into reminiscence therapy programs (13, 24). However, some limitations still existed in the current research: (1) the status of anxiety and depression was measured by one assessment (HADS scale); the following study should use other scales to improve the validation; (2) the intervention period was 12 months, which was relatively short; subsequent studies should conduct a long-term intervention; and (3) only patients who received surgical resection were enrolled; thus, our conclusion was not suitable for the unresectable HCC patients.

In conclusion, reminiscence therapy represents an effective treatment to mitigate anxiety and depression in and improve the quality of life of elderly patients with HCC.

## Data Availability

The original contributions presented in the study are included in the article/supplementary material, further inquiries can be directed to the corresponding author/s.
